# Antimicrobial, antibiofilm, cytotoxicity, and anti-DNA topoisomerase activity of *Streptomyces* sp. 22SH with ADME and in silico study

**DOI:** 10.1186/s12866-025-03912-w

**Published:** 2025-04-16

**Authors:** Mervat G. Hassan, Mohamed O. Abdel-Monem, Al Shaimaa M. A. Sleem, Mohamed E. El Awady, Ahmed A. Hamed

**Affiliations:** 1 Botany and Microbiology Department, Faculty of Science, Benha Univ., Benha, Egypt; 2 Microbial Biotechnology Department, National Research Centre, El-Buhouth St. 33, Dokki-Cairo, 12622 Egypt; 3 Microbial Chemistry Department, National Research Centre, El-Buhouth St. 33, Dokki-Cairo, 12622 Egypt

**Keywords:** *Streptomyces* sp., Bioactive metabolites, Antibacterial, Antibiofilm, anti-DNA topoisomerase

## Abstract

The genus *Streptomyces* has been recently proven to be a valuable and rich source of producing several bioactive compounds with substantial biological activity and applications in many fields such as medicine, environmental science, food industries, and agronomy. This study highlights the importance of Streptomyces as an antimicrobial, antibiofilm, and anticancer. Out of the 75 actinobacteria isolated from both marine and soil habitats, one isolate, HG2, was selected based on its potent antimicrobial activity. The isolate has been identified morphologically by studying colony and spore chain morphology using TEM and genetically by sequencing their 16 sr RNA gene as Streptomyces sp. 22SH with Accession number OK326829.1. Bioassay-guided fractionation of the Streptomyces sp. 22SH crude extract led to the isolation and purification of Cis-9-Octadecenoic. Biological evaluation including antimicrobial and antibiofilm activity of the crude and purified compound was performed on four clinical microbes (S. aureus ATCC6538-P, B. subtilis ATCC6633, and P. aeruginosa ATCC27853). The compound showed the ability to eradicate the biofilm formation by the tested pathogens. Additionally, the antitumor activity was assessed, and the compound showed a cytotoxic effect against liver carcinoma and breast cancer cells, with IC50 values of 17.48 ± 0.94 and 88.73 ± 4.78 µg/ml, respectively. While it displayed anti-topoisomerase activity with an IC_50_ of 0.65 ± 0.023 µg/ml. Furthermore, the compound’s ADME-related physicochemical features and docking analysis were investigated.

**Supplementary Information:**

The online version contains supplementary material available at 10.1186/s12866-025-03912-w.

## Introduction

Antimicrobial resistance (AMR) is the main cause of the public health disaster caused by antibiotic misuse in recent decades [1]. According to the World Health Organization, pathogenic diseases have become more difficult to cure, and death rates have risen due to antibiotic resistance (AR). New sources of antibiotics are urgently needed to combat these superbugs lying within the WHO priority list [2]. The financial impact of antimicrobial resistance (AMR) on national economies and health systems is significant because it makes patients or those who care for them less productive due to extended hospital admissions and the need for more expensive and intense treatment [3].

The number of patients whose treatments fail or who pass away from infections will rise in the absence of efficient instruments for the avoidance and effective management of drug-resistant illnesses and enhanced access to current and novel dependable antimicrobials. Surgery, such as hip replacements or cesarean sections, chemotherapy for cancer, and organ transplants, will all become more dangerous medical procedures (WHO, 2022) [4].

Coastal habitats are among the most productive and valuable habitat types in ecosystems around the globe [5]. Since they are home to creatures unique to the marine environment, marine ecosystems have proven to be a good source of innovative and distinctive natural products (NPs). Marine NPs are a valuable source for drug discovery since certain characteristics of the chemical diversity reported from marine organisms align with those of recognized medications [6]. Today, more marine medications are being used. Another 23 marine NPs are currently undergoing clinical studies, while 14 marine NPs or their derivatives are already approved as medications [7].

Actinomycetes, gram-positive filamentous bacteria, function as saprophytes and break down intricate biopolymers [8]. Actinobacteria, particularly those from the genus *Streptomyces*, are among the most significant producers of antibiotics. For example, *Streptomyces* species have been responsible for the discovery of clinically important antibiotics like streptomycin, tetracycline, and erythromycin, which are widely used to treat bacterial infections [9]. Given the increasing problem of germs that are resistant to several drugs, the search for novel antibiotics derived from actinobacteria is very critical. Moreover, actinobacteria have produced a number of anticancer chemicals, such as anthracyclines, which are used in cancer treatment because they may stop DNA production and cause cancer cells to die. Actinobacteria have the unusual capacity to synthesis a wide range of bioactive compounds, which emphasizes its potential to improve cancer and infectious disease therapies [10].

Additionally, they can create a variety of secondary metabolites, many of which have antibacterial or antifungal characteristics. The current study aims to screen and examine the bioactive secondary metabolites with antimicrobial and anticancer activity of marine and terrestrial actinomycetes obtained from different sources in the Egyptian region.

## Materials and methods

### Sample collection

Samples were taken from two habitats in Egypt: marine habitats (Ain Sokhna sediment (29.6725° N, 32.3370° E), Ras Sedr sediment (29.5933° N, 32.7178° E), and Hurghada sea water (27.2579° N, 33.8116° E) and soil habitats (Mansoura, Dakahlia Governorate). Samples were collected, coded, and transferred to the Lab and kept in a fridge at the Microbial chemistry department till further processing [11].

### Isolation of marine actinomycetes

Serial dilution techniques were utilised in order to successfully isolate actinomycetes from samples taken from both marine and soil environments. Utilizing starch casein media for marine samples and starch nitrate agar medium for soil samples was the method of choice. Isolation of actinomycetes was accomplished by utilizing their distinctive morphological traits, which included deep-sitting colonies, sporulation, and a colour that was easily identifiable.

### Biological screening of isolated actinomycetes small-scale extracts

To prepare the small-scale crude extract of the isolated actinomycetes, 75 actinomycetes isolates were cultured on rice media for 10 days at 30 °C. After incubation, each separate culture medium was mixed with ethyl acetate. The isolates’ crude extracts were then assessed for their antimicrobial potential toward a range of test pathogens using the agar disc diffusion method using nutrient agar media and potato dextrose agar with incubation period 24 h for bacteria and 48 h for fungi toward a panel of test microbes including gram-positive (*Staphylococcus aureus* ATCC6538-P subsp. *aureus*), a gram-negative bacterium (*Escherichia* coli ATCC 14169), and fungi (*Aspergillus niger* NRRL A-326). All test microbes were collected from Egypt’s National Research Center, Egypt [12].

### Identification of the most potent isolate (HG2)

#### Phenotypic identification and genotypic identification

The most potent isolate, HG2, showed a pronounced antimicrobial activity and has been identified by studying its morphological, physiological, and biochemical characteristics HG2’s colony formation on various growth mediums, color, textures, shapes, and sizes, and microscopic investigation of cellular features such as hyphal arrangements and spore generation revealed its morphology [13–16]. Identity confirmation of the selected isolate was carried out via sequencing of the 16 S rRNA gene. Genomic DNA was extracted and amplified using the following primers: Reverse 27 F (5′-AGAGTTTGATCCTGGCTCAG-3′) and forward 1492R (5′-TACGGYTACCTTGTTACGACTT-3′). The sequencing process made use of the Big Dye terminator cycle sequencing kit (Applied BioSystems, USA). The sequencing results were then evaluated using the Applied Bio-Systems model 3730XL, an automated DNA sequencing machine (Applied BioSystems, USA). The obtained 16 S rRNA sequence was then analyzed by aligning it using the BLAST online tool with other 16 S rRNA sequences at the GenBank, National Center for Biotechnology Information (NCBI) database. Bacterial strains with the highest similarity to our isolate’s 16 S rRNA gene were chosen, aligned, and used to build a phylogenetic tree.

### Large scale fermentation

To prepare the large-scale crude extract of the *Streptomyces* sp. 22SH, the spore suspension of the isolate was prepared by inoculation of the isolate into 100 mL of the ISP2 medium and then cultivated at 30 °C for three days. The cultivated seed culture was then used to inoculate 10 × 1 L Erlenmeyer flasks with 100 g of commercial rice and 100 mL of 50% seawater pH 6.5. After that, the inoculated cultures were incubated for 14 days at 28 °C [17]. The rice culture was then extracted with ethyl acetate (ratio 1:3) before being filtered, and concentrated in vacuo. The crude extract was then dried down to yield 4.5 gram.

### Bioassay guided fractionation and identification of isolated compound

Four grams of the obtained crude extract were placed on a normal-phase silica column with a diameter of seven centimeters. The adsorbent (silica gel) to solute (crude extract) ratio is 20:1. A total of 100 fractions of 5 mL each were gathered and subjected to a thin-layer chromatography (TLC) analysis. TLC was performed on Silica Gel 60 F254 (layer thickness 0.2 mm, E. Merck, Darmstadt, Germany) precoated TLC plates with Dichloromethane (DCM): methanol (90:10, v/v) as the solvent system. Based on the TLC results, the obtained similar fractions were recombined after confirmation by Ultra Violet and anisaldehyde/sulfuric acid reagent [18]. Biological evaluation of the obtained fractions was carried out using a variety of test microorganisms, including gram-positive bacteria *Staphylococcus aureus ATCC6538-P subsp. aureus* and *Bacillus subtilis* ATCC6633, and a gram-negative bacteria *Pseudomonas areuginosa* ATCC 27,853 and *Escherichia coli* ATCC25955, and fungi *Candida albicans* ATCC10231.

### Structural elucidation

LCMS uses liquid chromatography to separate chemicals and mass spectrometry to identify and quantify them by mass-to-charge ratio. Analyzing metabolites, small compounds, and peptides in complicated combinations is routine. NMR analyzes atomic nuclei’s interaction with a magnetic field and radiofrequency pulses to identify chemical structures. Its chemical shift and coupling data identify functional groups, molecular dynamics, and 3D structures, making it crucial for structural elucidation and conformational analysis. The most potent fraction was then submitted to Sephadex LH-20 column and the obtained fractions were biologically evaluated. The purified molecule was identified by measuring its molecular weight using Liquid Chromatography Mass Spectroscopy and Nuclear Magnetic Resonance (NMR).

### Biological evaluation of purified compound

#### Antibiofilm activity

To evaluate the antibiofilm activity of the obtained purified compound, an MTP assay was completed utilizing four clinical microorganisms (*P. aeruginosa* ATCC 27853, *S. aureus* ATCC6538-P, *E. coli* ATCC 25955, *and B. subtilis* ATCC6633) [19]. The study included inoculating sterile 96-well plates with overnight bacterial suspensions in nutrient-rich broth and adding the chemical at conc of 10 µg/mL. We rinsed wells with PBS to eliminate planktonic cells after 24 h of biofilm development at 37 °C, then stained with 0.1% crystal violet solution for 15 min. Removed excess stain, washed, and air-dried wells before solubilizing dye with ethanol. We assessed biofilm inhibition using 570 nm optical density (OD).

#### Cytotoxic effect

The antitumor activity was assessed at the Regional Center for Microbiology and Biotechnology, Al-Azhar University, Cairo, Egypt. The cell lines (HepG2 malignancy cells from the liver and the MCF7 cell line for breast cancer) were purchased from the American Type Culture Collection (ATCC, Rockville, MD). The Cells were cultured in appropriate media and incubated under standard conditions (37 °C, 5% CO₂). Serial dilutions of test compounds were prepared, and cells were exposed to varying concentrations for 24–72 h. Staurosporine was a positive control, while untreated cells acted as negative controls. Cell viability was assessed using the MTT assay with 5 mg/mL MTT reagent. After incubation, formazan crystals were dissolved in DMSO, and absorbance was measured at 570 nm with a reference wavelength of 630 nm. The half-maximal inhibitory concentration (IC₅₀) was calculated using dose-response curves, representing the concentration required to inhibit 50% cell viability. Staurosporine was used as a drug control agent [20].

#### DNA topoisomerase Inhibition activity

To measure the relaxation of supercoiled pBR322 plasmid DNA The extracted compound was used in a slightly altered version of a previously described procedure [21]. Etoposide was used as a drug control. Briefly, 0.5 µg of supercoiled pBR322 plasmid DNA was combined with a reaction buffer containing Tris-HCl, KCl, MgCl₂, and DTT. Topoisomerase I enzyme was added with varying concentrations of the test compound (1, 5, and 10 µM) or etoposide (10 µM) for the control. Reactions were incubated at 37 °C for 30 min, then stopped by adding loading dye. Samples were resolved on a 1% agarose gel stained with ethidium bromide and visualized under UV light.

### In silico forecasts prediction of toxicity and physicochemical characteristics associated with ADME

SwissADME web tools (https://www.swissadme.ch/) accessed (June 2024) were used to plan the obtained physicochemical properties of a compound’s ADME characteristics [22]. Using the ProTox ii web server, compounds’ in-silico toxicity predictions were carried out as previously published [23].

### The molecular docking

Outer membrane proteins (OMPs) of gram-negative bacteria are crucial in mediating bacterial virulence and antibiotic resistance, which in turn affect how harmful the bacteria are. Proteins in the outer layer and the OMPX (1QJ8) crystal structure were obtained from the Protein Data Bank (PDB) database and refined by removing water molecules and heteroatoms, followed by hydrogen atom addition to ensure stability. Energy minimization was conducted using MOE to optimize the receptor’s conformation. The *cis*-9-Octadecenoic acid structure was retrieved from PubChem in SDF format, converted to PDB via the https://cactus.nci.nih.gov. and subjected to energy minimization and geometric optimization before docking analysis. The prepared PDB files of both the receptor and ligand were uploaded to the PatchDock server, with receptor-ligand interaction mode set to default parameters. A root mean square deviation (RMSD) cutoff of < 4.0 Å was applied to refine the docking results. The highest-scoring docked complex was selected based on the PatchDock algorithm, and further interaction analysis was conducted in MOE. Residues within 4 Å of the ligand-binding site were examined to identify key molecular interactions, including hydrogen bonding, hydrophobic contacts, and electrostatic interactions.

## Results

### Isolation of streptomycetes and pre-screening

Seventy-five streptomycetes isolates were isolated from various marine and soil habitats, including Hurghada marine sea, Ras Sedr sediment, and Ain Sokhna sediment. While the soil sample was collected from Mansoura. *Streptomyces* sp. is isolated based on distinctive colony morphology, typically forming circular, convex-shaped colonies. Then, the isolated streptomycetes were cultivated on a rice medium for small-scale fermentation to obtain the bioactive compounds. Therefore, the antimicrobial activity of ethyl acetate extracts for isolated *Streptomyces* spp. was tested using the agar disc diffusion method against *E. coli* ATCC 14,169, *S. aureus* ATCC6538-P and *A. niger* NRRLA-326. Ciprofloxacin and Amphotericin B were used as controls. Among all tested actinomycetes, 20 isolates exhibited antimicrobial activity, including HG2, HG3, HG5, HG9, HG12, RS8, RS9, RS10, RS11, RS12, RS15, AS8, AS9, AS23, AS24, M1-2, M1-4, M1-7, M1-9, and M2-13. Out of the 20 isolates, one strain, HG2, exhibited potent antimicrobial activity (supplementary tables S1–S5).

### Phenotypic identification of most potent streptomycetes isolate HG2

**Fig. 1:**
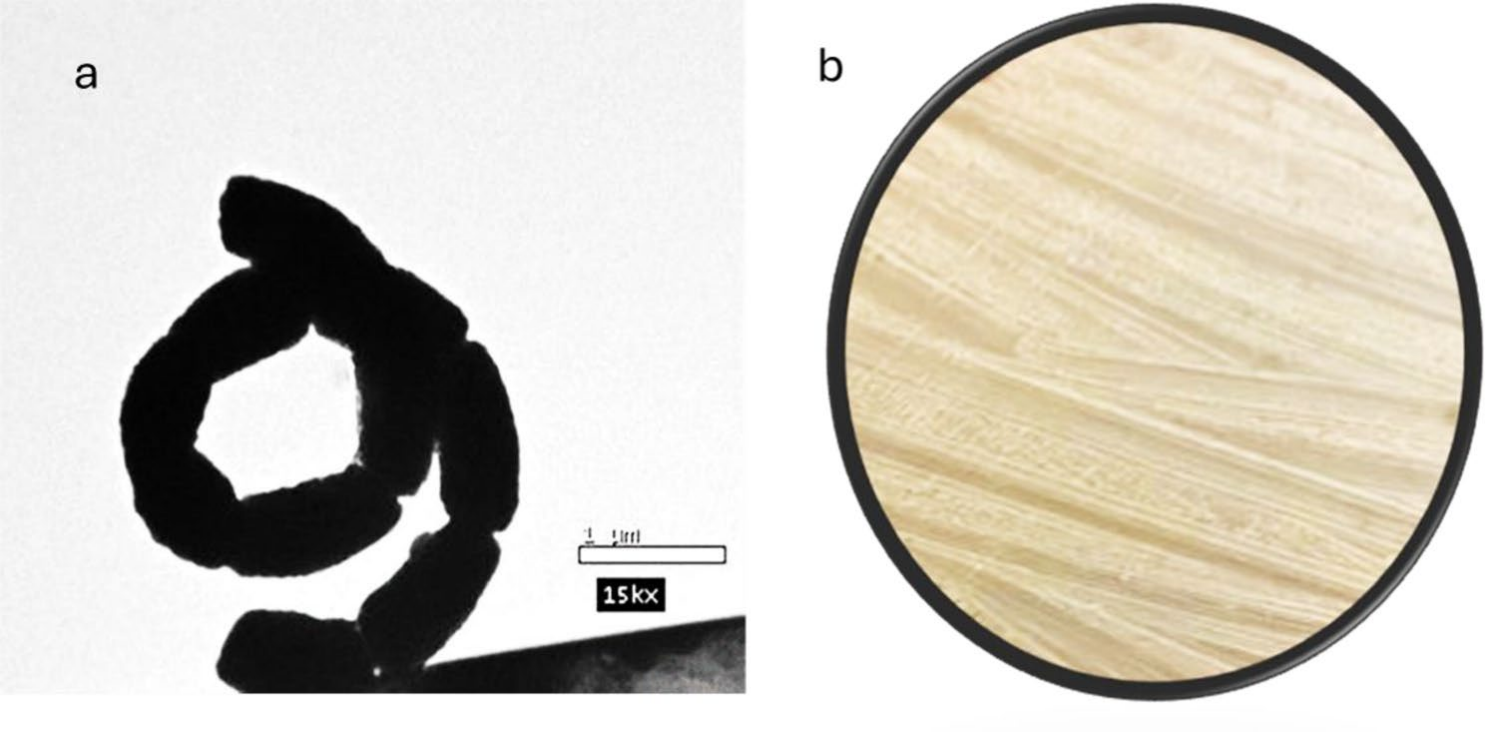
**a** TEM photomicrograph showing smooth spore surface (isolate HG2 × 15000). **b** colony morphology

To formally describe a novel taxon, actinobacteria are currently defined using a polyphasic approach that includes a combination of phenotypic, chemotaxonomic, and genotypic data. The primary components that need to be collected and examined in prokaryote characterization studies were described [24]. Therefore, the strain (HG2) was identified primarily by visualizing its spore morphology using TEM, and the obtained result showed that the strain has rectiflexible spore chains with a smooth spore surface, according to spore chain morphology Figure 1. Tyrosine agar does not produce melanin pigments in most cases. The spore mass is whitish and produces yellow diffusible pigments. The isolate HG2 demonstrates unique pigmentation patterns upon several media. In the starch nitrate medium, the aerial mycelium manifested as reddish grey, accompanied by greyish yellow substrate mycelium, but other media, including starch-ammonium sulfate and glycerol-asparagine, displayed grey aerial mycelium and yellowish grey substrate, indicating diversity in pigment synthesis (Table 1). Physiological and chemo-taxonomical studies are presented in Table 2. The obtained results showed that the isolated HG2 was able to produce melanin pigment only on tyrosine agar, while no melanin pigment was observed on peptone iron agar. The enzymatic capabilities of the isolate were also looked into, and the findings indicated that the isolate HG2 has proteolysis and lecithinase activity. The utilization of different carbon sources was also assessed, and results showed that the selected isolate HG2 can utilize D glucose, L Arabinose, and D fructose, while it can’t consume Rhamnose, Sucrose, D-xylose, D-mannitol, I-inositol, Galactose, and Raffinose.

**Table 1:** Cultural properties of isolate no. HG2 grown on different culture media

Medium	Color of		
	Aerial mycelium	Diffusible pigments	Substrate mycelium
1- Starch nitrate medium	Reddish gray	+ve	Grayish yellow
2- Starch-ammonium sulphate medium	Gray	-ve	Yellowish gray
3- Glycerol-asparagine medium	Gray	-ve	Yellowish gray
4- Oat-meal medium	Pale gray	-ve	Yellowish gray
5- yeast/malt extract agar medium	Whitish Gray	-ve	Pale gray
6- Czapeks medium	Gray	-ve	Pale gray

**Table 2:** Physiological and chemo-taxonomical properties of the isolate HG2

Isolate no.		HG2
Melanin pigment production	Pepton iron agar	-
	Tyrosine agar	+
Enzyme activities	Proteolysis	+
	Lipolysis	-
	Lecithinase	+
Utilization of different carbon source	No suger (-)	+
	D-Glucose (+)	+
	D-Fructose	+
	Sucrose	-
	Rhamnose	-
	D-Mannitol	-
	D-Xylose	-
	Raffinose	-
	I-inositol	-
	Galactose	-
	L-Arabinose	+
Nitrate reduction		+
H2S production		-
Starch hydrolysis		+
Cellulose decomposition		-
Gelatin liquification		+

### Genotypic identification of most potent streptomycetes isolate HG2

**Fig. 2:**
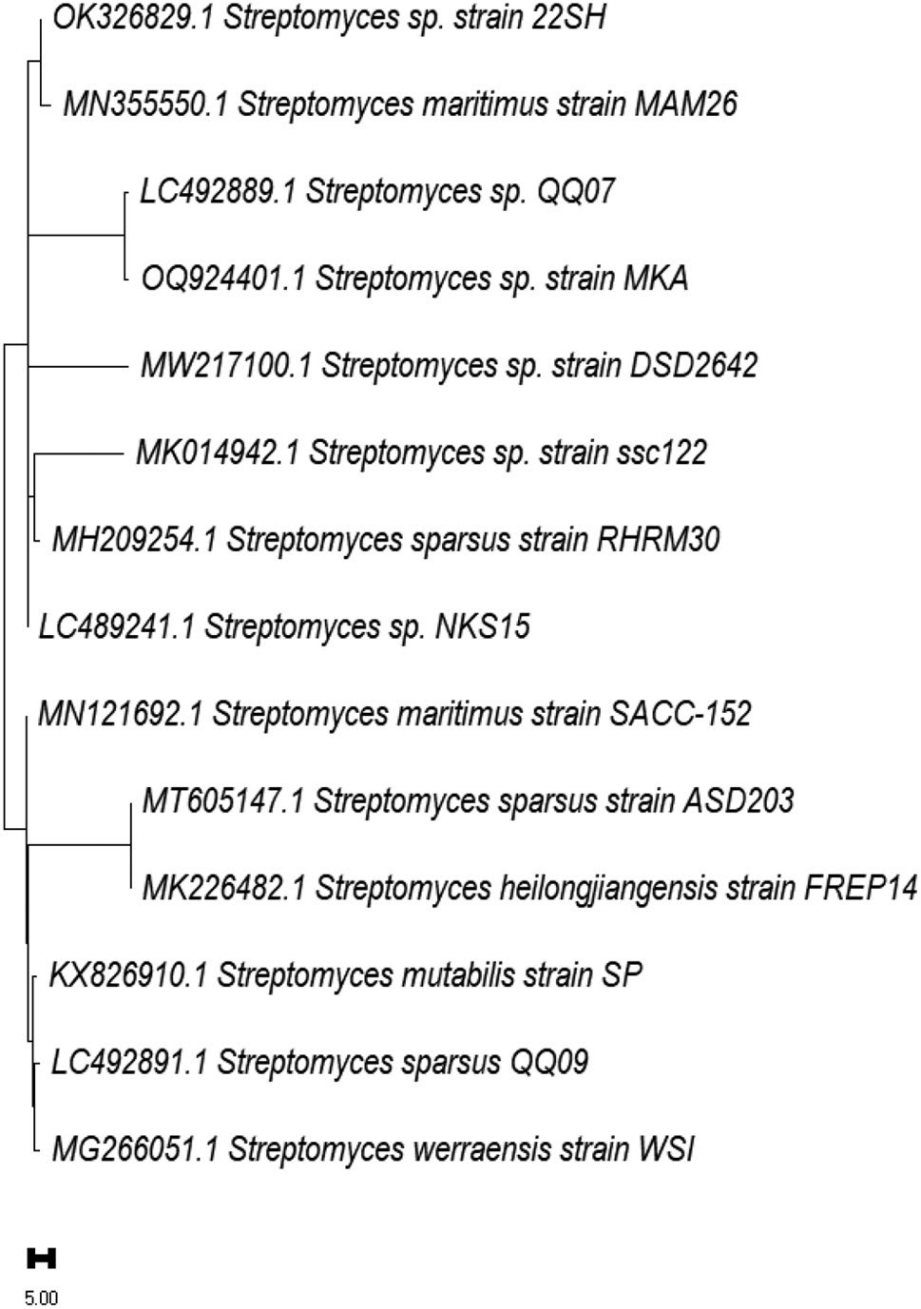
Phylogenetic tree of the *Streptomyces* sp. 22SH strain (Accession no. OK326829.1)

Analysis of the 16 S rDNA and sequencing of the most potent isolate *Streptomyces* isolate (HG2), started by isolating genomic DNA, and amplification. Sequencing and analysis of the Genomic DNA of the most potent isolate showed a high similarity score of isolate HG2, with *Streptomyces* sp., reaching 100% similarity. The *Streptomyces* sequence was submitted to the Gene Bank as *Streptomyces* sp. 22SH (Accession number: OK326829.1). The phylogenetic tree was constructed using MEGAX and presented in Figure 2.

### Large-scale production and purification of bioactive substances from *Streptomyces* sp. 22SH

The strain *Streptomyces* sp. 22SH was grown in a rice medium with ethyl acetate used for the extraction. After evaporation, the ethyl acetate was evaporated, and the obtained extract was 4.5 gram. The obtained extract was principally separated into 100 fractions by using flash column chromatography. All fractions were chemically screened using TLC. Based on the TLC screening, the 100 fractions were recombined into 14 fractions. The 14 obtained fractions were biologically screened by evaluation of their antimicrobial activity (Table 3). According to the antimicrobial results, the greatest active proportion (F2) was purified through a Sephadex LH-20 column with a DCM: Methanol gradient mobile phase. The TLC and antimicrobial evaluation of the obtained Sephadex fractions showed that only six fractions exhibited antimicrobial activity (Table 4). Among them, the subfraction (SF15) was the most potent, as it showed broad-spectrum activity toward all tested microbes. The most potent Sephadex subfraction (SF15) was further purified to remove any impurities, and the obtained semi pure compound was structurally identified using LC-MS and NMR.

**Table 3:** Antimicrobial activity of *Streptomyces* sp. 22SH flash column fractions

Extracts	Antibacterial activity (mm)				Antifungal activity (mm)
	Gram–ve		Gram + ve		
	*E. coli* ATCC25955	*P. aeruginosa* ATCC27853	*B. subtilis* ATCC6633	*S. aureus* ATCC6538-*P*	*C. albicans* ATCC10231
Crude	NA	13.00 ± 0.12	14.50 ± 0.21	10.00 ± 0.13	13.5 ± 0.12
F1	NA	11.00 ± 0.14	12.50 ± 0.12	9.9.00 ± 0.15	NA
F2	12 ± 0.02	23.00 ± 0.35	25.00 ± 0.15	22.00 ± 0.09	18.00 ± 0.13
F3	NA	15.00 ± 0.21	NA	NA	10.00 ± 0.09
F4	9 ± 0.12	NA	12.00 ± 0.07	NA	9.90 ± 0.06
F5	NA	NA	8.25 ± 0.06	NA	13 ± 0.12
F6	NA	NA	NA	NA	NA
F7	NA	NA	NA	NA	NA
F8	NA	NA	14.00 ± 0.12	NA	NA
F9	NA	NA	11.00 ± 0.23	NA	NA
F10	NA	NA	NA	NA	NA
F11	NA	14.00 ± 0.08	NA	NA	NA
F12	NA	NA	NA	NA	NA
F13	NA	NA	NA	NA	NA
F14	NA	NA	NA	NA	NA
Strep.	17.2	24. 6	25.3	23.8	-
Amp	-	-	-	-	22.9

NA: Not active Strep.: Streptomycin Amp: Amphotericin B

**Table 4:** Antimicrobial activity of *Streptomyces* sp. 22SH Sephadex fractions

Extracts	Antibacterial activity (mm)				Antifungal activity (mm)
	Gram–ve		Gram + ve		
	*E. coli* ATCC25955	*P. aeruginosa* ATCC27853	*B. subtilis* ATCC6633	*S. aureus* ATCC6538-*P*	*C. albicans* ATCC10231
Crude	NA	14.00 ± 0.23	13.50 ± 0.03	11.00 ± 0.12	15.00 ± 0.11
SF1	NA	10.00 ± 0.06	NA	7.00 ± 0.03	NA
SF2	7.00 ± 0.12	10.00 ± 0.12	NA	NA	NA
SF3	12.00 ± 0.20	13.00 ± 0.12	12.00 ± 0.01	10.00 ± 0.14	8.50 ± 0.12
SF4	11.00 ± 0.03	NA	11.00 ± 0.03	NA	9.90 ± 0.13
SF5	NA	NA	12.00 ± 0.09	NA	13.00 ± 0.23
SF6	NA	NA	10.00 ± 0.06	NA	NA
Strep.	17.2	24. 6	25.3	23.8	-
**Amp**	**-**	**-**	**-**	**-**	**22.9**

NA: Not active Strep.: Streptomycin Amp: Amphotericin B

The Molecular weight (282.47) and Molecular formula (C_18_H_34_O_2_) of the isolated pure compound were measured using LC-MS. The H-NMR spectrum of the compound demonstrated signs at δ 0.83 as a pair of triplets overlapped, suggesting two methyl groups at the terminals at δ 1.29 as a broad singlet for a long chain of methylene protons, at δ 1.94 for methylene groups α to C = C group, and at δ 2.24 for methylene groups α to carbonyl group. The multiple signals were visible at δ 4.13, which may be attributed to the oxymethylene group, and at δ 5.38 for an unsaturated proton. Consequently, the compound could be identified as cis-9-Octadecenoic (C1) depending on its chromatographic characteristics, proton (Supplementary S6) [25].

**Figure:**
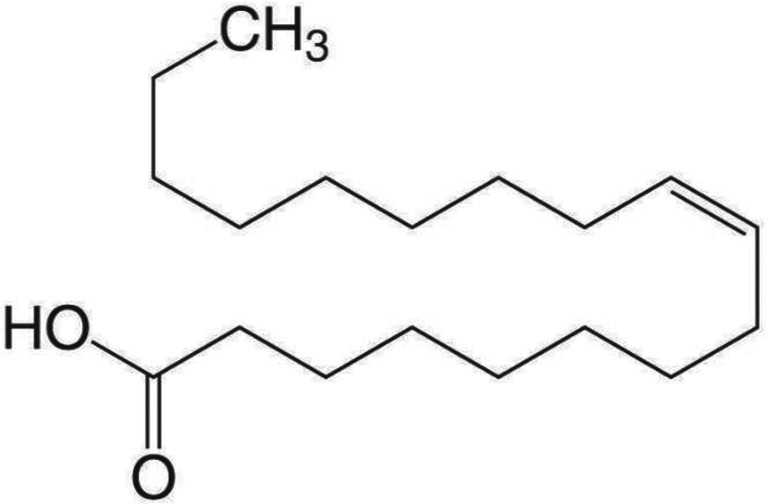
C1. Cis-9-Octadecenoic acid

### Biological evaluation of identified compound C1

#### Antibiofilm activity

**Fig. 3:**
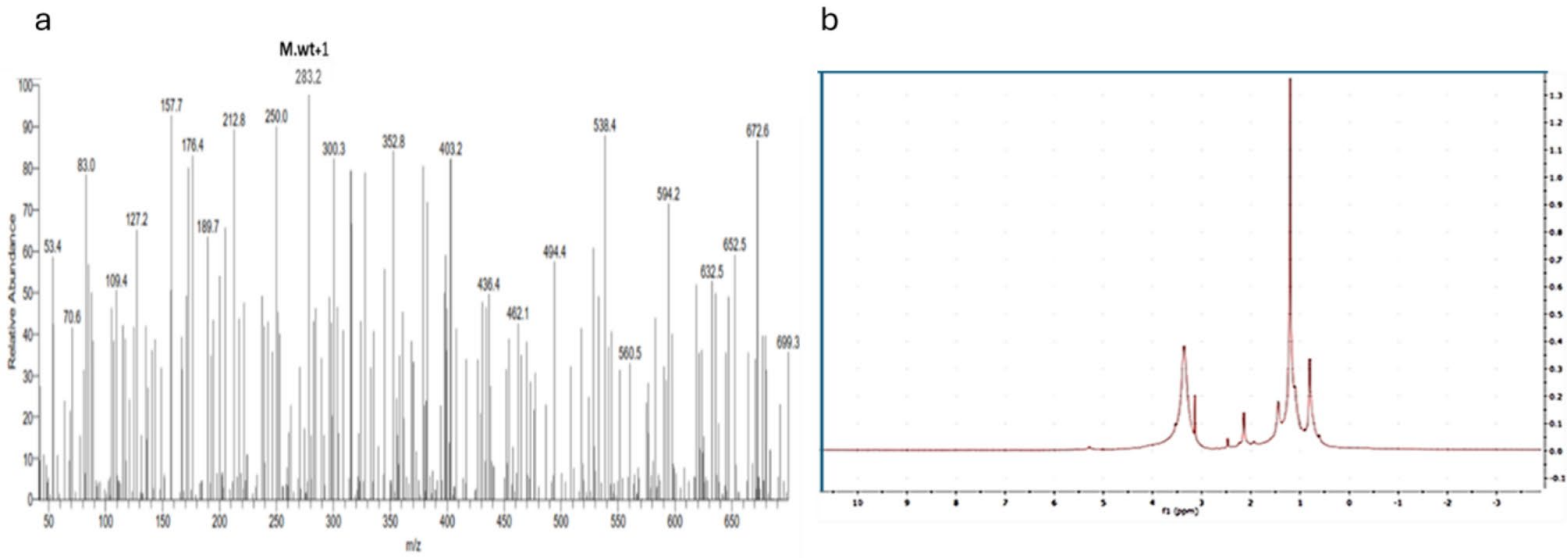
**(a)** Mass spectrum of cis-9-Octadecenoic (**b**) NMR proton diagram of semi pure fraction contain cis-9-Octadecenoic

The anti-biofilm activity of C1, MTP assay was carried out using four clinical microbes (Fig. 3). It showed antibiofilm activity against *S. aureus* ATCC6538-P, *B. subitilis* ATCC6633 and *P. aeruginosa* ATCC27853 with inhibition ratios of 30.50, 50.00, and 45.26%, respectively. While it showed no antibiofilm activity against *E. coli* ATCC25955.

#### Cytotoxic activity

**Fig. 4:**
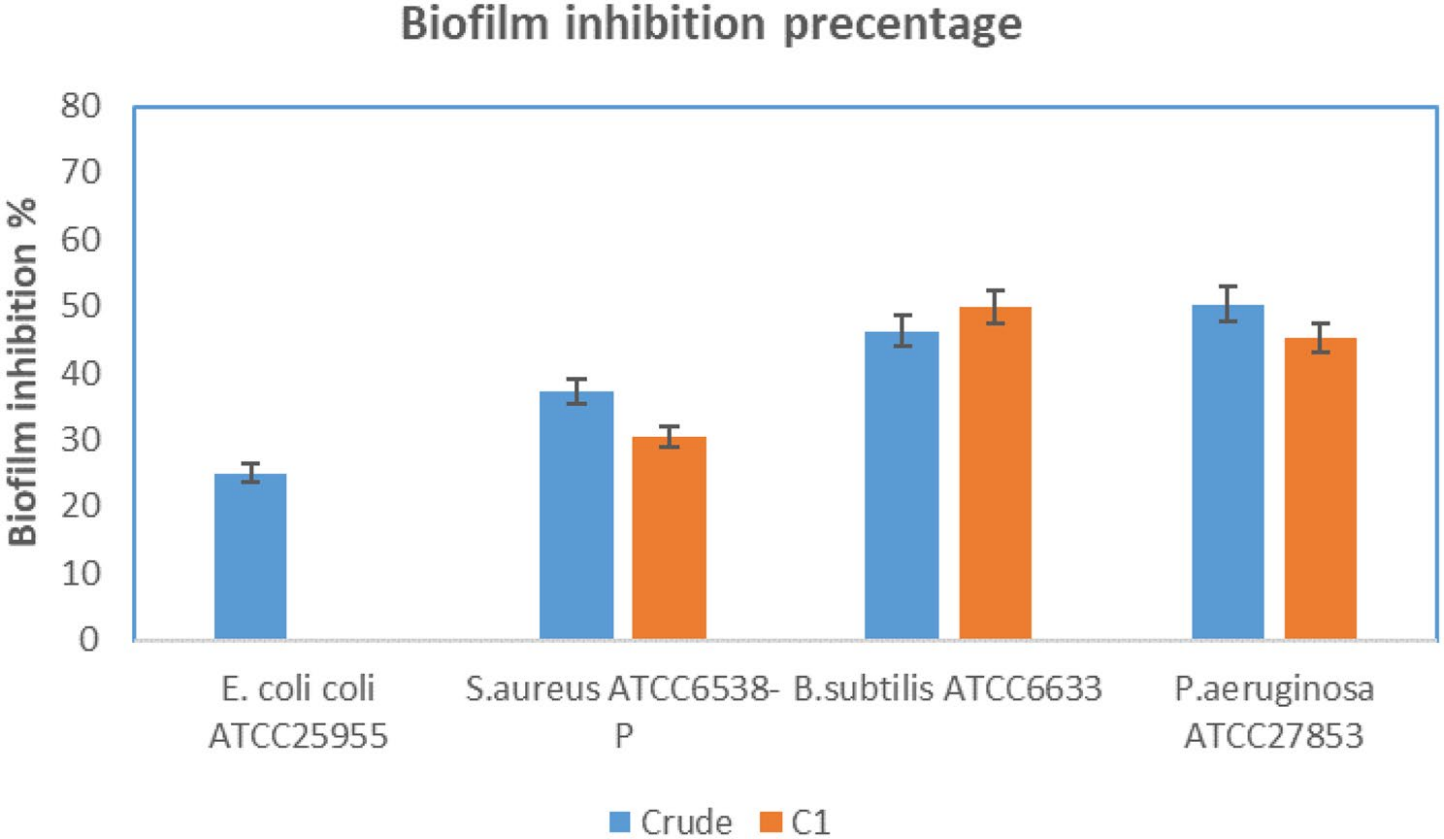
Biofilm inhibition of obtained compound C1

The anticancer activity of the identified compound was assessed and results showed that C1 has a pronounced anticancer effect against liver carcinoma with an IC_50_ value of 17.48 ± 0.94 µg/mL while it showed a moderate anticancer effect against breast cancer cells with an IC_50_ value of 88.73 ± 4.78 µg/ml (Fig. 4). Compared to the standard, the semi-pure compound significantly reduced MCF7 and HepG2 viability at 100 µM (log 2) to 44.26% and 35.67%, respectively, demonstrating robust anticancer activities. Cell viability rose with decreasing concentrations, peaking at 82.93% and 85.24% at the lowest dose (log 0.409), reaching 79.33% (MCF7) and 74.67% (HepG2) at 1.56 µM (log 0.1931). The results show dose-dependent cytotoxicity, with more potent effects at higher concentrations (Supplementary S7).

#### DNA topoisomerase Inhibition activity

**Fig. 5:**
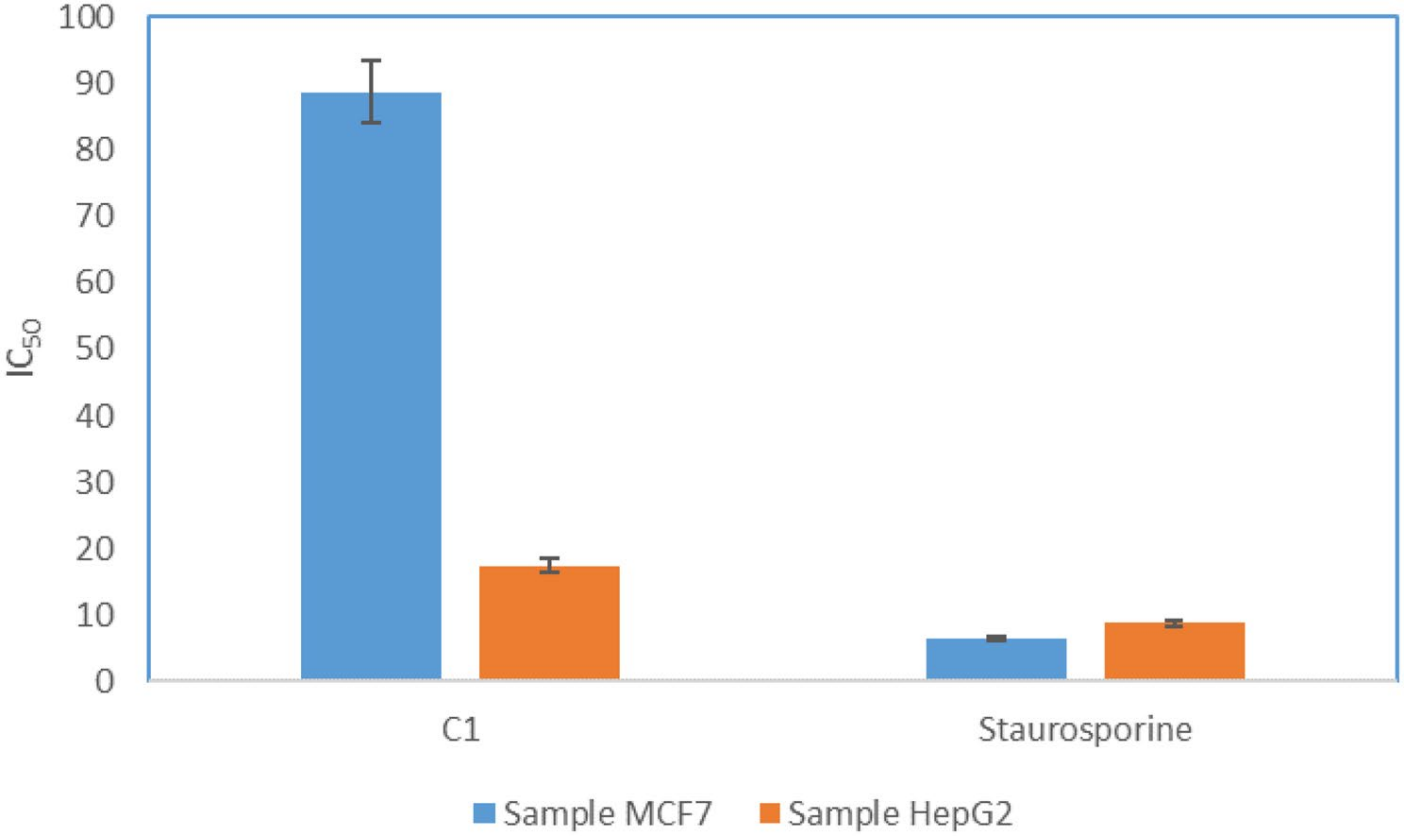
Anticancer activity of obtained compound C1

DNA Topoisomerase inhibition assay was performed for cis-9-Octadecenoic, which showed anti-topoisomerase activity with an IC_50_ 0.65 ± 0.023 ug/ml, while Etoposide gave 1.87 ± 0.07 µg/ml (Fig. 5).

### The ADME-related physicochemical properties

**Fig. 6:**
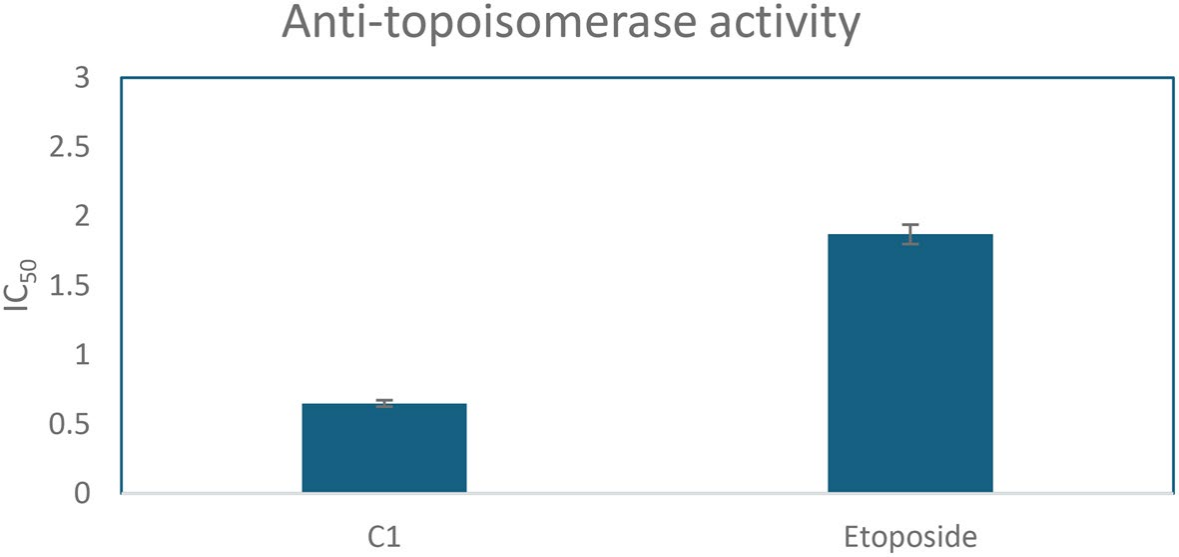
DNA Topoisomerase inhibition activity

The physicochemical characteristics of the ADME of the cis-9-Octadecenoic acid were measured, and the obtained data showed that the compound exceeded the Lipinski requirements with 1 violation (MLOGP > 4.15) but did not pass the Veber rules with 1 violation (1 violation: Rotors > 10), while one violation (WLOGP > 5.6) has been discovered using the Ghose rule. The substances have a 0.85% oral bioavailability and may be taken as a pill (Supplementary S8). Additionally, by plotting bioavailability, a quick evaluation of drug similarity was carried out. The radar image of the cis-9-Octadecenoic acid according to the six physicochemical traits size, lipophilicity, polarity, solubility, saturation, flexibility, and the pink region shows the parameter’s ideal value range. Using the obtained diagram as a guide, the compound shows the optimum (pink area) for all the parameters except the flexibility parameter (Fig. 6a). The cis-9-Octadecenoic acid showed Log P_o/w_ values above 5 (5.71), suggesting weak permeability and absorption throughout the cell membrane. Based on the ESOL topological model, the tested compound is moderately soluble. Regarding defining medicinal chemistry and Lead likeness, the compound property failed to meet the requirement of three (RO3), as it has two violations for this rule (Rotors > 7, XLOGP3 > 3.5). The compound had moderate synthetic accessibility, with values of 3.07 for the synthetic accessibility score (SAscore), which calculates accessibility based on fragment similarity and complexity penalties. Employing the vector machine algorithm (SVM) model, the pharmacokinetic characteristics of cis-9-octadecenoic acid were determined [22]. The Compound demonstrated a specific inhibition effect on CYP1A2 and CYP2C9 isoenzymes, while no selective activity against CYP2C19, CYP2D6, and CYP2C9 isoenzymes has been detected (Supplementary S8). Figures 6b indicates the boiled-egg model (Brain or Intestinal Estimate D permeation method, WLOGP vs. TPSA), which was modified from [22], The cis-9-Octadecenoic acid showed significant gastric (GI) absorption in humans. The cis-9-Octadecenoic acid are non-P-gp substrates (PGP-, red dots), while their blood-brain barrier (BBB) is permeant (TPSA < 75 Å²), indicating the possibility of central nervous system (CNS) effects [26]. The skin permeability prediction coefficient (Kp) of cis-9-Octadecenoic was completed as outlined by Potts and Guy [27]. The cis-9-Octadecenoic showed a log (Kp) (-2.60 cm/scm/s), while the compounds with a high negative log Kp had less skin penetration. The white region (GI) indicates a very high probability of HIA (GI) absorption, while the yellow zone (yolk) indicates a very possible BBB permeability. The gray area outside represents substances that have no brain penetration and a poor absorption rate. Additionally, the points are labeled red if P-GP non-substrate (PGP) is expected and blue if P-gp substrate (PGP+) is anticipated. While forecasting the toxicity of the obtained cis-9-Octadecenoic acid was performed using the ProTox ii webserver, Results in Supplementary (S8) showed that the obtained compound acts on certain targets as expected by ProTox ii. While Supplementary (S9) shows the toxicity radar chart, which is meant to quickly demonstrate the certainty of positive toxicity data in comparison to the class average.

### Molecular Docking determines the cis-9-Octadecenoic acid binding capacity to *E. coli*

**Fig. 7:**
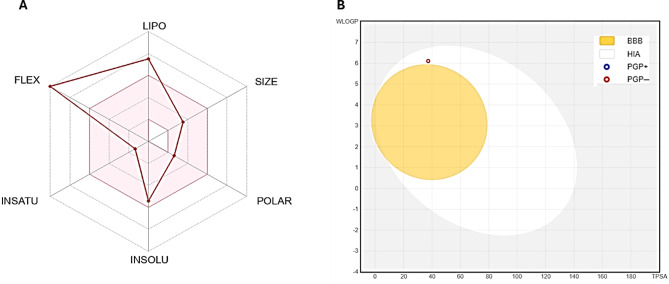
**A** Bioavailability radar char and **B** boiled-egg plot

A computational study and molecular docking of the possible interaction and antimicrobial activity of cis-9-Octadecenoic acid was carried out using MOE software and Patch Dock server for cis-9-Octadecenoic acid and the three-dimensional structures of *E. coli* proteins in the outer membrane, the OMPX (1QJ8) crystal structure. The interactions between the amino acid residues and cis-9-Octadecenoic and the kinds of interactions between Cis-9-Octadecenoic, and OMPX (1QJ8) reacted via Phe90, and Lys48 could result in disruption of the *E. coli* outer membrane (Fig. 7a, b and c). The binding energy between *cis*-9-octadecenoic acid and OmpX was calculated to be -5.2 kcal/mol. Several reports study the OmpX inhibition by streptomyces compounds. In the study conducted by Basharat et al. [28], Eleven compounds were analyzed, with seven satisfying Lipinski’s Rule of Five, indicating favorable drug-like properties. The highest binding energies identified were in the range of -10 kcal/mol. These inhibitors interact with OmpX by forming hydrogen bonds, hydrophobic interactions, and Van der Waals forces, primarily engaging key residues such as ASP97 and ARG171. Structural changes in the active pocket upon ligand binding highlight the inhibitors’ stability and effectiveness. The findings suggest that these seven compounds hold promise as potential therapeutic agents targeting OmpX-related diseases. The findings highlight how cis-9-octadecenoic acid could be a promising molecule for destabilizing bacterial membranes through specific residue targeting, advancing our understanding of its antimicrobial mechanism.

## Discussion

*Streptomyces* species can produce many different biologically active secondary metabolites, such as antibiotics, pesticides, antiparasitic, herbicides, and enzyme inhibitors, which are very important therapeutically and commercially. About one-third of known antibiotics are extracted from *Streptomyces sp.*. Researchers are very interested in the potential of *Streptomyces* species to create secondary metabolites [29]. This capability is typically a result of the clusters of *Streptomyces* strains, which are responsible for encoding enzymes for numerous secondary metabolic processes [30]. Nevertheless, dietary variables and growth circumstances are also important regulators of secondary metabolite formation in microorganisms [31]. Out of 75 actinomycetes isolates purified from marine and soil habitats one actinomycetes coded as HG2, was selected as it exhibited potent antimicrobial activity. Several studies conducted by researchers showed that actinomycetes have exhibited obvious antimicrobial activities against almost all tested pathogenic strains. Chen et al. [32] isolated *Streptomyces* sp. K15 and discovered that a few of its secondary metabolites have strong antibacterial properties. Marine *Streptomyces aminophilus* exhibited activity against almost all test organisms and high activity against *Aspergillus niger*. The literature reported that this strain can produce many bioactive compounds with variable activities, such as chorismic acid, a macrolide antibiotic, and a potent immunosuppressant [33–35]. Additionally, the marine *Streptomyces baarnensis* exhibited moderate activity against *Pseudomonas aeruginosa*,* Staphylococcus aureus*,* Candida albicans*, and *Saccharomyces cerevisiae*, high activity against *Aspergillus niger*, and no activity against *Bacillus subtilis*. This strain produced many antibiotics, such as enaminomycin A, B, and C [36]. Two methods including morphological and genetic—identified the isolate. Polyphasic morphological, chemotaxonomic, and genotypic data are used to define actinobacteria as a novel taxon [38]. In prokaryote characterization research, major components must be gathered and evaluated. Thus, the strain (HG2) was identified by examining its spore shape using TEM, which displayed rectiflexible spore chains with a smooth spore surface. Most tyrosine agar does not create melanin. Spore mass is pale and produces yellow diffusible colors. Table 2 lists physiological and chemo-taxonomical investigations. The isolated HG2 produced melanin pigment only on tyrosine agar, not peptone iron. HG2 exhibits proteolysis and lecithinase activity, according to enzymatic analysis. The isolate HG2 can use D glucose, L Arabinose, and D fructose, but not Rhamnose, Sucrose, D-xylose, D-mannitol, I-inositol, Galactose, or Raffinose. 16 S rDNA analysis and sequencing of the most potent Streptomyces isolate (HG2) began with genomic DNA isolation and amplification. Genomic DNA sequencing and analysis of the most potent isolation indicated 100% similarity between isolate HG2 and *Streptomyces* sp. The Gene Bank received *Streptomyces* sp. 22SH (Accession number: OK326829.1). To obtain the isolate crude extract, the strain was cultivated in rice media, incubated and extracted with ethyl acetate. The obtained crude extract was then partially purified using flash column chromatography and reassembled after TLC analysis into 14 fractions. Out of the assembled fractions only six fractions exhibited antibacterial activity. The most potent subfraction, SF15, demonstrated wide-ranging effectiveness against all bacteria. Additional purification removed contaminants from the most potent Sephadex subfraction (SF15). The NMR and LC-MS structural characterization of the molecule revealed its identity as cis-9-Octadecenoic (a mono-unsaturated omega-9 fatty acid) that can be found in a variety of sources, both plant and animal. Triglyceride esters of Cis-9-Octadecenoic comprise the majority of olive oil. In pharmaceuticals and aerosol goods, Cis-9-Octadecenoic acid is utilized as an emulsifying or solubilizing ingredient. It may slow the growth of the fatal condition of adrenoleukodystrophy, which damages the brain and adrenal glands, and it may improve memory. Cis-9-Octadecenoic acid may also be responsible for the Olive oil’s hypotensive (or blood pressure-lowering) properties [37]. Antimicrobial resistance, which is currently recognized as a significant health issue, is closely associated with pathogenic bacterial biofilm. This biofilm serves as a crucial element in bacterial pathogenicity and is a fundamental factor in the development of persistent illnesses caused by various bacteria. Scientists have discovered the presence of an extracellular polymeric substance (EPS) that envelops biofilms, which are intricate formations composed of polysaccharides, proteins, and DNA. Biofilm formation, characterized by an acidic pH and reduced oxygen levels, significantly contributes to antibiotic resistance due to decreased drug diffusion and penetration [39]. The antibiofilm activity of the identified compound was evaluated against four clinical microbes and results showed antibiofilm activity appeared toward S. *aureus* ATCC6538-P, *B. subitilis* ATCC6633 and *P. aeruginosa* ATCC27853 with inhibition ratios of 30.50, 50.00, and 45.26%, respectively. While it showed no antibiofilm activity against *E. Coli* ATCC25955. Additionally, the anticancer activity of the identified compound showed that a pronounced anticancer activity is present against liver carcinoma with an IC_50_ value of 17.48 ± 0.94 µg/mL while it showed a moderate anticancer effect against breast cancer cells with an IC_50_ value of 88.73 ± 4.78 µg/ml. Several studies revealed an induced reduction in cell proliferation by cis-9-Octadecenoic acid among many tumor cell lines. Herein, the well-known oncogene HER2 (erbB-2), which is important for the genesis, invasive growth, and metastasis of several human malignancies, could be suppressed by cis-9-Octadecenoic acid. Cis-9-Octadecenoic acid might be involved in proliferation-related intracellular calcium signaling pathways. Regarding cell death, cis-9-Octadecenoic acid has been demonstrated to cause cell death in cancer cells. The processes via which apoptosis is triggered by cis-9-Octadecenoic acid could be related to an increase in intracellular ROS generation or caspase-3 activity. According to reports, several unsaturated fatty acids trigger apoptosis by releasing calcium from intracellular reserves. However, there is insufficient data to support such a role for cis-9-Octadecenoic acid [39]. Topoisomerase II is an enzyme necessary for chromosomal condensation, DNA replication, and chromosome segregation. Inhibitors of Topoisomerase II are crucial treatments used to treat a variety of neoplasms, such as lymphomas, sarcomas, breast cancer, lung cancer, and testicular cancer [40]. Recent molecular studies showed that inhibition of Topoisomerase II is a successful cancer chemotherapy plan. The molecular tools that have allowed an understanding of the biological functions of Topoisomerase II are also being used to learn the specifics of how drugs work. According to this research, topoisomerase II can be precisely targeted as a potent anticancer method [41]. A DNA Topoisomerase inhibition experiment was conducted for cis-9-Octadecenoic, revealing anti-topoisomerase activity with an IC50 of 0.65 ± 0.023 ug/ml. In comparison, Etoposide exhibited a concentration of 1.87 ± 0.07 µg/ml. In order to comprehend and forecast the behavior of a molecule throughout the body, encompassing its absorption, distribution, metabolism, and excretion, an analysis was conducted on the ADME physicochemical and pharmacokinetic properties of cis-9-Octadecenoic acid. In addition, the ProTox ii website was utilized to assess the toxicity profile of cis-9-octadecenoic acid. In addition, a computational analysis was conducted to investigate the potential interaction and antimicrobial effects of cis-9-Octadecenoic. This analysis utilized the MOE software and Patch Dock server to examine the three-dimensional structures of E. coli proteins found in the outer membrane, specifically the crystal structure of OMPX (1QJ8). The interaction between Cis-9-Octadecenoic and OMPX (1QJ8) through Phe90 and Lys48 may lead to the breakdown of the outer membrane of *E. coli*. Several studies have shown the biological and agricultural potential of marine-derived actinomycetes, notably Streptomyces. Streptomyces violaceusniger KS20 has many biomedical uses, according to Chakraborty et al. [42], while Math et al. found that 7-hydroxyflavone from *Amycolatopsis* sp. HSN-02 can control Cercospora leaf spot disease in tomatoes [43]. *Streptomyces* filamentous strain KS17, from underexplored maritime environments, inhibited human infections with its broad-spectrum antibacterial action [44]. *Streptomyces* levis strain KS46’s secondary metabolite profile showed potential in marine settings [45]. Thes researches demonstrate the role actinomycetes’ role in producing novel antimicrobials and sustainable biocontrol methods.

## Conclusion

In conclusion, our study emphasizes the importance of investigating marine microbial diversity as a significant resource bioactive compound. The bioactive metabolites obtained from streptomyces sp. exhibited potent antibacterial and antibiofilm properties against *S. aureus*, *B. subtilis*, and *P. aeruginosa*, effectively disrupting biofilm formation. Furthermore, it demonstrated significant cytotoxicity against liver and breast cancer cells, with IC₅₀ values of 17.48 ± 0.94 and 88.73 ± 4.78 µg/ml, respectively, alongside vigorous anti-topoisomerase activity (IC₅₀ = 0.65 ± 0.023 µg/ml). Computational analysis, including ADME profiling and molecular docking, provided insights into its pharmacokinetic potential. These findings underscore *cis*-9-Octadecenoic acid as a promising candidate for antimicrobial and anticancer applications. However, our study has limitations. The compound’s efficacy and safety must be confirmed in vivo after in vitro testing. Computational research revealed pharmacokinetics, but experimental studies are needed to validate. Future study should enhance fermentation conditions and how the molecule is manufactured to increase yield and effectiveness.

**Fig. 8:**
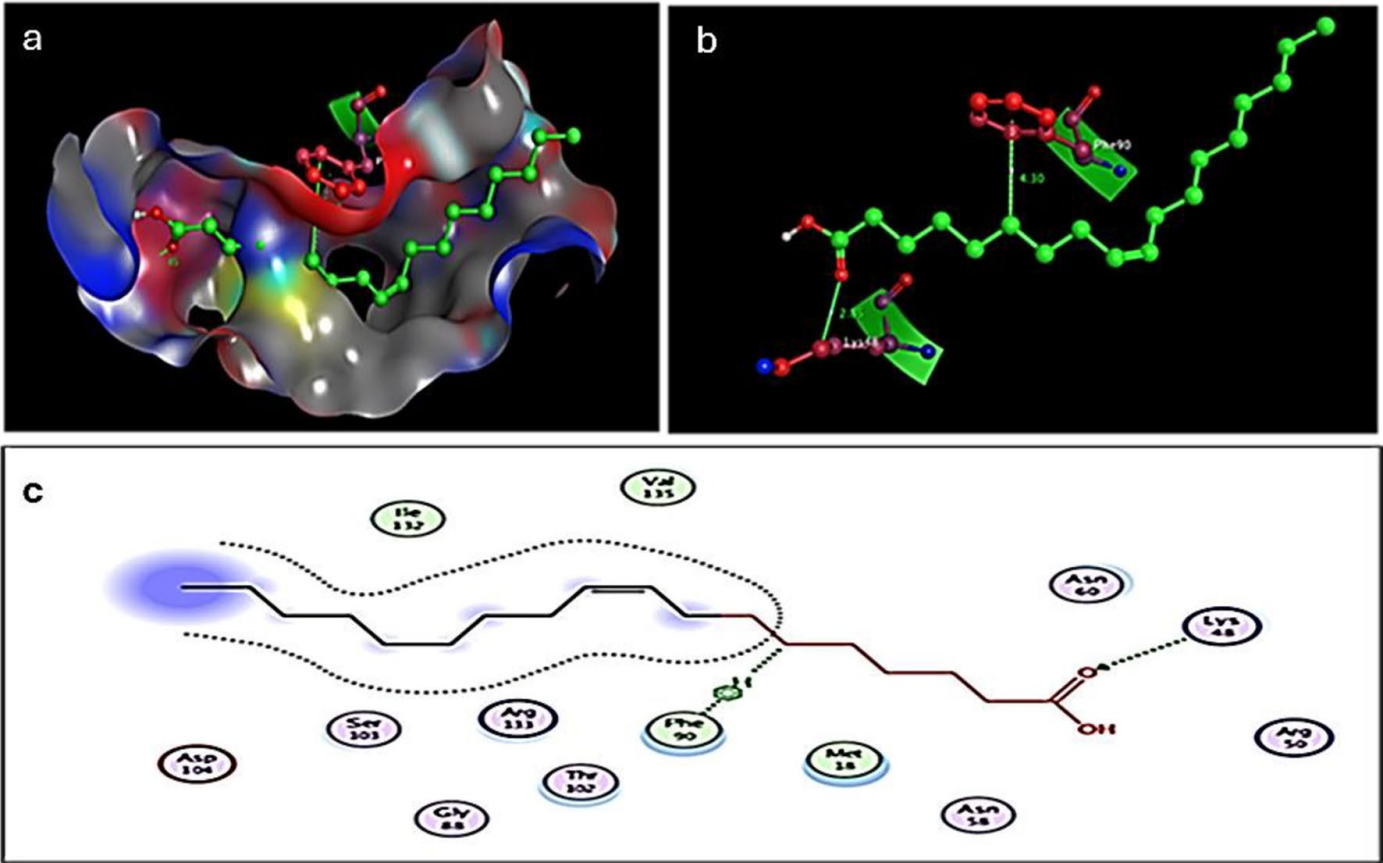
Molecular docking determines cis-9-Octadecenoic binding capacity to *E. coli* outer membrane protein. (**a** and **b**) The docking model shows an interaction of the cis-9-Octadecenoic with 1QJ8 through Phe90, and Lys48, (**c**) 2D Interaction

## Electronic supplementary material

Below is the link to the electronic supplementary material.

Supplementary Material 1

## Data Availability

Sequence data that support the findings of this study have been deposited in the National Center for Biotechnology Information with the primary accession code OK326829.
