# Supplementary material for: Antimicrobial, antibiofilm, cytotoxicity, and anti-DNA topoisomerase activity of Streptomyces sp. 22SH with ADME and in silico study
Source: BMC Microbiol. 2025 Apr 16;25:219. doi: 10.1186/s12866-025-03912-w (PMC12001559; doi:10.1186/s12866-025-03912-w)

**Antimicrobial, antibiofilm, cytotoxicity, and anti-DNA Topoisomerase activity of *Streptomyces* sp. 22SH with ADME and *in silico* study**

Mervat G. Hassan^1^, Mohamed O. Abdel-Monem^1^, Al Shaimaa M. A. Sleem^1^, Mohamed E. El awady^2^, Ahmed A. Hamed^3*^

^1^Botany and Microbiology Department, Faculty of Science, Benha Univ., Benha, Egypt.

^2^Microbial Biotechnology Department, National Research Centre, El-Buhouth St. 33, Dokki-Cairo 12622, Egypt. <https://orcid.org/0000-0001-5155-3949>

^3^Microbial Chemistry Department, National Research Centre, El-Buhouth St. 33, Dokki-Cairo 12622, Egypt.

^*^Corresponding author: Ahmed A. Hamed, Mervat G. Hassan

E-mail: [ahmedshalbio@gmail.com](mailto:ahmedshalbio@gmail.com), [mervat.hassan@fsc.bu.edu.eg](mailto:mervat.hassan@fsc.bu.edu.eg)

**Supplementary (S1). Antimicrobial activity for *streptomyces* ethyl acetate extracts isolated from sea water Hurgada**

| **Extracts from isolate no.** | **Antimicrobial activity (clear zone, mm)** | | |
| --- | --- | --- | --- |
|  | ***E. coli***  **ATCC 14169** | ***S. aureus***  **ATCC6538-P** | ***A. niger***  **NRRL A-326** |
| **HG1** | NA | NA | NA |
| **HG2** | 15.7± 0.10 | 15.9± 0.32 | 14.8± 0.16 |
| **HG3** | NA | 17.4± 0.46 | 13.9± 0.13 |
| **HG4** | NA | NA | NA |
| **HG5** | 12.25± 0.17 | 14.8± 0.5 | 14.3± 0.17 |
| **HG6** | NA | NA | NA |
| **HG7** | NA | NA | NA |
| **HG8** | NA | NA | NA |
| **HG9** | 10.5± 0.25 | 12.4± 0.13 | 10.9± 0.12 |
| **HG10** | NA | NA | NA |
| **HG11** | NA | NA | NA |
| **HG12** | NA | 11.4± 0.12 | 11.8± 0.23 |
| **HG13** | NA | NA | NA |
| **HG14** | NA | NA | NA |
| **HG15** | NA | NA | NA |
| **Cip.** | 17.0± 0.25 | 12.0± 0.18 | - |
| **Amp.** | - | - | 23.6± 0.31 |

**NA:** Not active  **Amp:** Amphotericin B

**Supplementary (S2). Antimicrobial activity for *Streptomyces* ethyl acetate extracts isolated from Ras Sedr sediment**

| **Extracts from isolate no.** | **Antimicrobial activity (clear zone, mm)** | | |
| --- | --- | --- | --- |
|  | ***E. coli***  **ATCC 14169** | ***S. aureus***  **ATCC6538-P** | ***A. niger***  **NRRL A-326** |
| **RS1** | NA | NA | NA |
| **RS2** | NA | NA | NA |
| **RS3** | 10.3± 0.15 | 13.2± 0.52 | NA |
| **RS4** | 15.4± 0.22 | 10.2± 0.12 | NA |
| **RS5** | 12.5± 0.14 | 11.2± 0.16 | NA |
| **RS6** | 11.3± 0.24 | 13.2± 0.52 | 10.2± 0.52 |
| **RS7** | 16.3± 0.16 | 13.2± 0.62 | NA |
| **RS8** | NA | NA | NA |
| **RS9** | NA | NA | NA |
| **RS10** | 9.9± 0.13 | 10.6± 0.24 | NA |
| **RS11** | NA | NA | NA |
| **RS12** | NA | NA | NA |
| **RS13** | NA | NA | NA |
| **RS14** | NA | NA | NA |
| **RS15** | NA | NA | NA |
| **Cip.** | 17.0±0.25 | 12.0± 0.18 | - |
| **Amp.** | - | - | 23.6± 0.31 |

**NA:** Not active  **Amp:** Amphotericin B

**Supplementary (S3). Antimicrobial activity for *streptomyces* ethyl acetate extracts isolated from Ain Sokhna sediment**

| **Extracts from isolate no.** | **Antimicrobial activity (clear zone, mm)** | | |
| --- | --- | --- | --- |
|  | ***E. coli***  **ATCC 14169** | ***S. aureus***  **ATCC6538-P** | ***A. niger***  **NRRL A-326** |
| **AS1** | NA | 10.1± 0.21 | 5.3± 0.25 |
| **AS2** | NA | 14.7± 0.13 | 11.4± 0.26 |
| **AS3** | NA | NA | NA |
| **AS4** | NA | NA | NA |
| **AS5** | NA | NA | NA |
| **AS6** | NA | NA | NA |
| **AS7** | NA | NA | NA |
| **AS8** | NA | NA | NA |
| **AS9** | NA | NA | NA |
| **AS10** | NA | NA | NA |
| **AS11** | 11.5± 0.11 | 14.0± 0.15 | 15.0± 0.20 |
| **AS12** | 10.3± 0.15 | 13.0± 0.13 | 11.4± 0.25 |
| **AS13** | NA | NA | NA |
| **AS14** | NA | NA | NA |
| **AS15** | NA | NA | NA |
| **AS16** | NA | NA | NA |
| **AS17** | NA | NA | NA |
| **AS18** | NA | NA | NA |
| **AS19** | NA | NA | NA |
| **Cip.** | 17.0±0.25 | 12.0±0.18 | - |
| **Amp.** | - | - | 23.6± 0.31 |

**NA:** Not active  **Amp:** Amphotericin B

**Supplementary (S4). Antimicrobail activity for *streptomyces* ethyl acetate extracts isolated soil of Mansoura 1**

| **Extracts from isolate no.** | **Antimicrobial activity (clear zone, mm)** | | |
| --- | --- | --- | --- |
|  | ***E. coli***  **ATCC 14169** | ***S. aureus***  **ATCC6538-P** | ***A. niger***  **NRRL A-326** |
| **M1- 1** | NA | NA | NA |
| **M1- 2** | 10.6± 0.17 | 11.2± 0.15 | 11.2± 0.16 |
| **M1- 3** | NA | NA | NA |
| **M1- 4** | 12.8± 0.20 | 10.7± 0.18 | 10.3± 0.25 |
| **M1- 5** | NA | NA | NA |
| **M1- 6** | NA | NA | NA |
| **M1- 7** | 12.0± 0.21 | 10.9± 0.17 | 10.2± 0.12 |
| **M1- 8** | NA | NA | NA |
| **M1- 9** | 12.0± 0.23 | 10.3± 0.43 | 10.4± 0.26 |
| **M1- 10** | NA | NA | NA |
| **Cip.** | 17.0± 0.25 | 12.0± 0.18 | - |
| **Amp.** | - | - | 23.6± 0.31 |

**NA:** Not active  **Amp:** Amphotericin B

**Supplementary (S5). Antimicrobial activity for streptomycetes ethyl acetate extracts isolated from soil of Mansoura 2**

| **Extracts from isolate no.** | **Antidermatophytic activity (clear zone, mm)** | | |
| --- | --- | --- | --- |
|  | ***E. coli***  **ATCC 14169** | ***S. aureus***  **ATCC6538-P** | ***A. niger***  **NRRL A-326** |
| **M2- 1** | NA | NA | NA |
| **M2- 2** | NA | NA | NA |
| **M2- 3** | NA | NA | NA |
| **M2- 4** | NA | NA | NA |
| **M2- 5** | NA | NA | NA |
| **M2- 6** | NA | NA | NA |
| **M2- 7** | NA | NA | NA |
| **M2- 8** | NA | NA | NA |
| **M2- 9** | NA | NA | NA |
| **M2- 10** | NA | NA | NA |
| **M2- 11** | NA | NA | NA |
| **M2- 12** | NA | NA | NA |
| **M2- 13** | NA | 12.8± 0.22 | 11.6± 0.35 |
| **M2- 14** | NA | NA | NA |
| **M2- 15** | NA | NA | NA |
| **M2- 16** | NA | NA | NA |
| **Cip.** | 17.0±0.25 | 12.0±0.18 | - |
| **Amp.** | **-** | **-** | **23.6± 0.31** |

**NA:** Not active  **Amp:** Amphotericin B

**Supp**


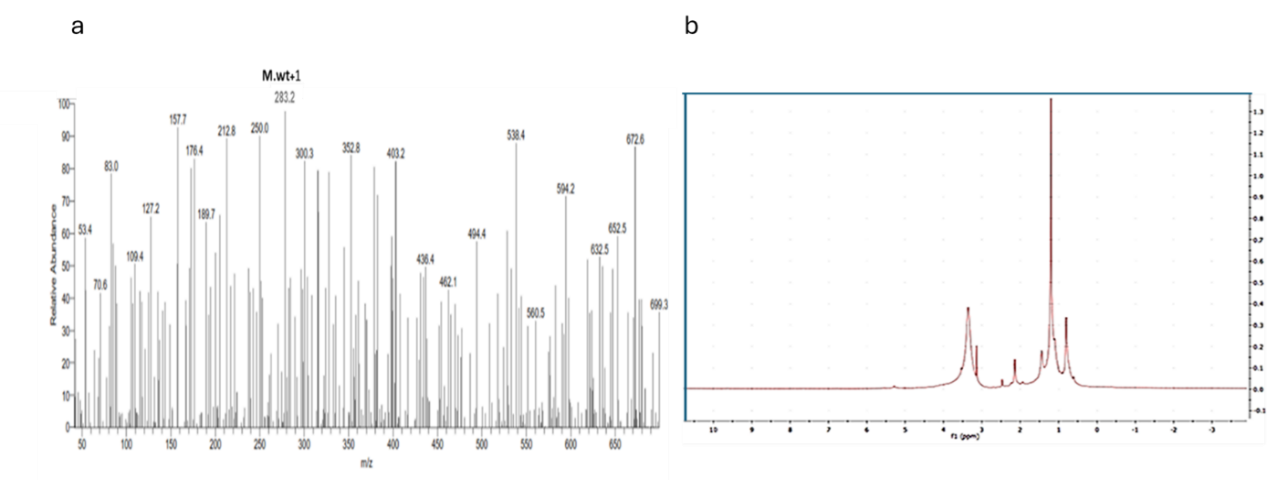


**Supplementary (S6). (a).**  Mass spectrum of cis-9-Octadecenoic (b). NMR proton diagram of semi pure fraction contain cis-9-Octadecenoic

**Supplementary (S7). Viability of the different cancers cell lines treated with different concentration**

|  | |  |  |  |
| --- | --- | --- | --- | --- |
| **log conc. (uM)** | **% Viability (MCF7(**  **(comp)** | **% Viability (MCF7) (Sta)** | **% Viability (HepG2( (comp)** | **% Viability ) HepG2( (Sta)** |
| **2** | **44.26** | **30.85** | **35.67** | **30.463** |
| **1.3979** | **64** | **37.865** | **45.73** | **41.1** |
| **0.7959** | **68.16** | **49.845** | **56.22** | **54.776** |
| **0.1931** | **79.33** | **62.197** | **74.67** | **60.709** |
| **0.409** | **82.93** | **70.267** | **85.24** | **76.483** |

**Supplementary (S8).** ADME-related physicochemical parameters of C1

| **parameters** | | **Values** |
| --- | --- | --- |
| **Physicochemical Properties** | M.Wt | 282.46 g/mol |
|  | Bonds in a molecule that can rotate | 15 |
|  | Atoms that can accept hydrogen bonds | 2 |
|  | Atoms that can donate hydrogen bonds | 1 |
|  | Molar Refractivity | 89.94 |
|  | Topological polar surface area (TPSA) | 37.30 Å² |
| **Lipophilicity** | Log Po/w (XLOGP3) | 7.64 |
|  | Log *P*_o/w_ (WLOGP) | 6.11 |
|  | Log *P*_o/w_ (MLOGP) | 4.57 |
| **Solubility** | Log *S* (ESOL) | -5.41 |
|  | Solubility | 3.03e-02 mg/ml ; 1.09e-04 mol/l |
|  | Class | Moderately soluble |
| **Druglikeness** | Lipinski (RO5) | Yes; 1 violation: MLOGP>4.15 |
|  | Ghose | No; 1 violation: WLOGP>5.6 |
|  | Veber | No; 1 violation:  Rotors>10 |
|  | Bioavailability Score | 0.85 |
| **Leadlikness** | Rule of three (RO3) | No; 2 violations: Rotors>7, XLOGP3>3.5 |
|  | Synthetic accessibility | 3.07 |
|  | GI (HIA) absorption | High |
|  | BBB permeant | No |
|  | P-GP substrate | No |
|  | CYP1A2 inhibitor | Yes |
|  | CYP2C19 inhibitor | No |
|  | CYP2C9 inhibitor | Yes |
|  | CYP2D6 inhibitor | No |
|  | CYP3A4 inhibitor | No |
|  | Log Kp (skin permeation: cm/s) | -2.60 cm/s |

**Supplementary (S9).** *In silico* toxicity prediction of C1

| **Classification** | **Target** | **Prediction** |
| --- | --- | --- |
| **Organ toxicity** | Hepatotoxicity | Inactive |
| **Toxicity endpoints** | Immunotoxicity | Inactive |
| **Toxicity endpoints** | Mutagenicity | Inactive |
| **Toxicity endpoints** | Cytotoxicity | Inactive |
| **Tox21-nuclear receptor signaling pathways** | Aryl hydrocarbon Receptor (AhR) | Inactive |
| **Tox21-nuclear receptor signaling pathways** | Androgen Receptor (AR) | Inactive |
| **Tox21-nuclear receptor signaling pathways** | Androgen Receptor Ligand Binding Domain (AR-LBD) | Inactive |
| **Tox21-nuclear receptor signaling pathways** | Aromatase | Inactive |
| **Tox21-nuclear receptor signaling pathways** | Estrogen Receptor Alpha (ER) | Inactive |
| **Tox21-nuclear receptor signaling pathways** | Estrogen Receptor Ligand Binding Domain (ER-LBD) | Inactive |
| **Tox21-nuclear receptor signaling pathways** | Peroxisome Proliferator-Activated Receptor Gamma (PPAR-Gamma) | Active |
| **Tox21-Stress response pathways** | Nuclear factor (erythroid-derived 2)-like 2/antioxidant responsive element (nrf2/ARE) | Active |
| **Tox21-Stress response pathways** | Heat shock factor response element (HSE) | Active |
| **Tox21-Stress response pathways** | Mitochondrial Membrane Potential (MMP) | Inactive |
| **Tox21-Stress response pathways** | Phosphoprotein (Tumor Suppressor) p53 | Inactive |
| **Tox21-Stress response pathways** | ATPase family AAA domain-containing protein 5 (ATAD5) | Inactive |

**Supplementary (S9). Toxicity radar chart**


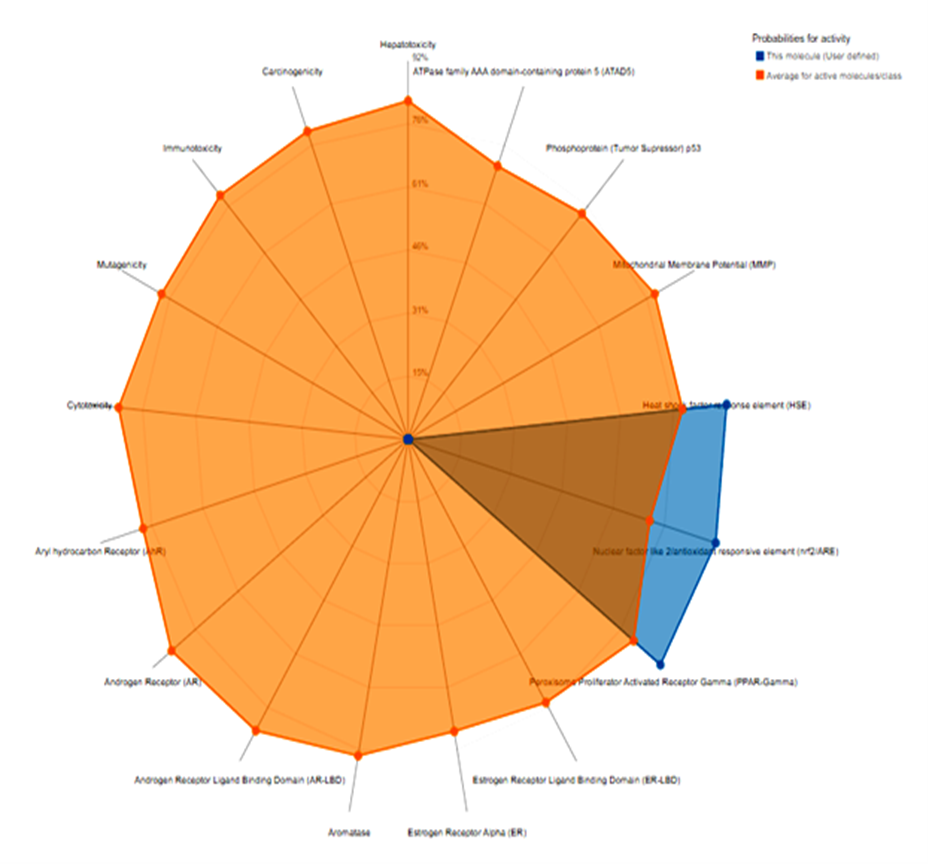

Supplement: Supplementary file 1 — Supplementary Material 1 [file 12866_2025_3912_MOESM1_ESM.docx]
